# Editorial: Microbiota biodiversity of traditional fermented products

**DOI:** 10.3389/fmicb.2024.1380205

**Published:** 2024-02-20

**Authors:** Biao Suo, Marcela Paola Castro, M. Y. Sreenivasa

**Affiliations:** ^1^College of Food Science and Technology, Henan Agricultural University, Zhengzhou, Henan Province, China; ^2^Laboratorio de Microbiología de Alimentos, Instituto de Investigaciones en Procesos Tecnológicos Avanzados (INIPTA-CONICET), Universidad Nacional del Chaco Austral (UNCAus), Sáenz Peña, Chaco, Argentina; ^3^Department of Studies in Microbiology, University of Mysore, Mysuru, India

**Keywords:** fermentation, microbial diversity, metagenomics, metaproteomics, health benefits, quality assessment

Fermentation stands as one of humanity's most extensively employed traditional techniques for processing, preservation, and introducing diversity to food products, imparting them with distinctive sensory and physical characteristics and extending their shelf life. The varied microbiota prevailing in fermented foods significantly contribute to fostering gut health through their involvement in digestion and nutrient absorption. Furthermore, the fermentation process produces bioactive compounds such as vitamins, peptides, organic acids, exopolysaccharides, amino acids and proteins which have been linked to improved immune function and overall wellbeing. However, despite their cultural significance and potential health advantages, there is a noticeable lack of comprehensive research on the microbial composition and health benefits of these traditional foods. Hence the research focus on “Microbiota diversity of traditional fermented products” was initiated to resonate within the scientific community encouraging further exploration of this uncharted territory by utilizing advanced research methodologies.

Baijiu is an alcoholic food with a long history in China, while its brewing process is extremely complicated. A correlation study on the dynamics of microbial community, physicochemical properties and volatile flavor compounds in the Xiasha round of the cave-brewed sauce-flavor Baijiu (CBSB) was reported by Ren et al. This study established the correlation networks and metabolic maps to understand the relationship between 18 dominant microbial genera and 23 significantly different volatile metabolites in the fermentation process. The investigation established theoretical foundations for CBSB, thereby creating a robust groundwork for subsequent craft optimization and the enhancement of Baijiu quality. Wu et al. conducted a parallel study utilizing multi-omics technology to examine the interplay between microbial community structure and volatile compounds in Cigar stacking fermentation. The study emphasized the interaction of diverse microbiota during the fermentation of various cigar varieties, which also impacted the development of aroma and other microbial agents. Further, Liu et al. conducted a study exploring the impact of extended fermentation time on the microbiota and wine quality in the fermentation process of Luxiang-flavor Baijiu. The research also analyzed the patterns of change in flavor substances and microbial flora.

The utilization of predictive functional profiles of microbial communities in fermented foods is an effective method for annotating the metabolic pathways within the gene sequences of microorganisms. Das et al. employed shotgun-based metataxonomic sequence analysis to report the microbial community structure and functional profiles of naturally fermented bamboo shoots. The study identified 49 phyla, 409 families, 841 genera, and 1,799 species, with Firmicutes as the dominant phylum (89.28%). Furthermore, it unveiled genes related to amino acid metabolism, pectin degradation, lipid metabolism, and other crucial pathways, suggesting their potential role in enhancing the nutritional and sensory qualities of fermented bamboo shoot products. In a metagenomic investigation, Sessou et al. contrasted bacterial diversity during the spontaneous fermentation of raw cow milk in two geographically distinct regions: Northeast India and West Africa. The study addressed the safety concerns in spontaneous milk fermentation, proposing strategies and technological interventions for controlled fermentation to ensure consistent, high-quality products with desired properties. Moreover, Xu et al. performed a meta-analysis using metagenomic sequencing data to investigate the potential benefits and risks associated with traditional fermented foods. The study underscored the impact of raw materials, regions, and substrates on microbial diversity and taxonomic composition, offering a thorough assessment of fermented food microbiomes. Another metagenomic investigation conducted by Lv et al. examined the microbial diversity of red vinasse acid, a traditional fermented food and identified key metabolic processes and essential enzyme genes related to sugar and amino acid metabolism. The study also delved into the role of dominant strains in primary metabolic pathways, confirmed the expression of crucial enzyme genes, and provided fundamental research data for the improvement of fermenting techniques to enhance the quality of red vinasse acid. Further, a metataxonomic analysis of indigenous and starter microbiota throughout the ripening process of Gouda cheese produced using unpasteurized milk contaminated with *Listeria monocytogenes* was carried out by Salazar et al. The study examined the bacterial community dynamics in Gouda cheese from unpasteurized milk, with and without *L. monocytogenes*, and identified notable differences in microbiomes during ripening. Unique taxa emerged in cheese without *L. monocytogenes* after 90 days, emphasizing the potential impact of *L. monocytogenes* on the microbial community. The findings underscored the importance of accounting for milk source characteristics in future metataxonomic studies on Gouda cheese.

Expanding upon metagenomic insights, metaproteomics delves into the functional aspects of microbial communities by examining the proteins expressed, providing a holistic understanding of the genetic potential uncovered through genomic analysis. The recently emerging data-independent acquisition (DIA) mass spectrometry in metaproteomics has proven valuable in gaining insights into the functions of microbiota, a domain constrained in genomic studies. Zhao et al. utilized quantitative metaproteomics to unveil the composition and metabolic characteristics of microbial communities in Chinese liquor fermentation starters, specifically Daqu. The outcome of the study highlighted unique microbial features in different Daqu types, with seasonal variations anticipated to guide the optimization of yield, quality, and flavor in liquor production

Another significant aspect of the microbiota biodiversity in traditional fermented products involves the strategies for inoculation, crucial for determining the flavor quality of fermented foods. In a study by Ye et al., the effects of the direct injection strategy and traditional inoculation strategy on physicochemical indices and flavor substances were investigated during the acetic acid fermentation process of Zhenjiang aromatic vinegar. The outcomes revealed that the direct inoculation strategy resulted in elevated levels of total acid, organic acid, and amino acid in comparison to the traditional inoculation approach. Additionally, it effectively stimulated acetoin production. While the traditional inoculation method demonstrated greater strain diversity, it exhibited lower relative abundance of major microbial genera throughout the fermentation process compared to the direct inoculation approach. Further a study by Pan et al. explored the impact of inoculating a single strain of *Bacillus licheniformis* and the microbiota comprising *B. velezensis* and *B. subtilis* on the microbial community and enzymatic activities of medium-temperature Daqu (MTD). The findings suggested that the bacterial community in MTD showed higher sensitivity to bioturbation than the fungal community, leading to variations in enzymatic activities. The indigenous microbiota exhibited a more pronounced response to the single strain than to the microbiota, augmenting the composition of functional microbiota related to liquefying activity (LA) and saccharifying activity (SA) in MTD.

Numerous scientific studies consistently demonstrate the potential health benefits of probiotics in humans. Given the global epidemic of diabetes and its rising association with western eating habits, there is a growing concern. In response, Huligere et al. conducted a study to assess the potential probiotic *Lactobacillus* spp. isolated from traditional fermented batters for potential use in diabetes treatment. The probiotic isolates displayed noteworthy probiotic properties, inhibitory activities against α-glucosidase and α-amylase indicating their potential as a source of anti-diabetic properties and, upon purification, could be employed as probiotic supplements. However, additional *in vivo* research is essential for comprehensive assessment.

Furthermore, a study by Bhutia et al. on the assessment of microbiological safety of traditionally processed fermented meat products was incorporated into the research theme. This study involved identifying potential spoilage or pathogenic bacteria, detecting enterotoxins, and screening antibiotic susceptibility patterns. Also, the study emphasized the need for proper hygiene monitoring during preparation to ensure the safety of these culturally accepted meat products.

In summary, the studies have delved into various facets of microbial diversification in fermented food products, demonstrating significant promise through metagenomic and metaproteomic analyses, as well as evaluations of potential health benefits and quality assessments.

## Author contributions

BS: Writing—review & editing. MC: Writing—review & editing. MS: Writing—original draft, Writing—review & editing.

